# Identification of the Methanogenesis Inhibition Mechanism Using Comparative Analysis of Mathematical Models

**DOI:** 10.3389/fbioe.2019.00093

**Published:** 2019-05-08

**Authors:** Magela Odriozola, Edo Abraham, Maria Lousada-Ferreira, Henri Spanjers, Jules B. van Lier

**Affiliations:** Department of Water Management, Delft University of Technology, Delft, Netherlands

**Keywords:** anaerobic membrane bioreactor (AnMBR), cationic polymer, flux enhancers, langmuir isotherm, methanogenesis inhibition, modeling, Monte Carlo (MC), specific methanogenic activity (SMA)

## Abstract

The application of cationic polymers to enhance membrane fluxes in anaerobic membrane bioreactors has been proposed by several authors. However, literature shows contradictory results on the influence of those chemicals on the biological activity. In this research, we studied the effect of a cationic polymer on the production of methane from acetate by acetoclastic methanogens. We assessed the effect of polymer concentration on the accumulated methane production (AMP) and the specific methanogenic activity (SMA) in batch tests. Batch tests results showed lower SMA values at higher concentrations of polymer and no effect on the final AMP. Different inhibition models were calibrated and compared to find the best fit and to hypothesize the prevailing inhibition mechanisms. The assessed inhibition models were: competitive (M1a), non-competitive (M2a), un-competitive (M3a), biocide-linear (M4a), and biocide-exponential (M5a). The parameters in the model related to the polymer characteristics were adjusted to fit the experimental data. M2a and M3a were the only models that fitted both experimental SMA and AMP. Although M1a and M4a adequately fitted the experimental SMA, M1a simulations slightly deviated from the experimental AMP, and M4a considerably underpredicted the AMP at concentrations of polymer above 0.23 gCOD L^−1^. M5a did not adequately fit either experimental SMA and AMP results. We compared models a (M1a to M5a), which consider the inhibition by the concentration of polymer in the bulk liquid, with models b (M1b to M5b) considering the inhibition being caused by the total concentration of polymer in the reactor. Results showed that the difference between a and b models' simulations were negligible for all kinetic models considered (M1, M2, M3, M4, and M5). Therefore, the models that better predicted the experimental data were the non-competitive (M2a and M2b) and un-competitive (M3a and M3b) inhibition models, which are biostatic inhibition models. Consequently, the decreased methanogenic activity caused by polymer additions is presumably a reversible process

## Introduction

Anaerobic membrane bioreactor (AnMBR) is an innovative technology for municipal wastewater treatment (Smith et al., 2012) and an established technology for industrial wastewater treatment with several full-scale AnMBRs already treating wastewater from food processing industries (Dereli et al., 2012). However, the deposition and accumulation of particles on the surface and in the membrane pores, generally designated as “fouling,” causes a reduction in the permeate flux. Lower fluxes translate into higher membrane surface requirements, which leads to increased construction costs, taking into account that membranes represent a significant amount in the total cost of an AnMBR (Lin et al., 2013). Therefore, fouling control is the main challenge in AnMBRs, with the low flux being the main factor limiting economic feasibility and applicability of this technology (Ozgun et al., 2013).

Several papers report the use of adsorbents, coagulants, and flocculants, usually called flux enhancers, to decrease membrane fouling and to increase the operational flux in membrane bioreactors. Cationic polymers, such as commercial synthetic polymers composed of Polydiallyldimethylammonium chloride (polyDADMAC), have been successfully used as flux enhancers in both aerobic and anaerobic membrane bioreactors. Koseoglu et al. (2008) tested three cationic polymers (MPL30, MPE50, KD452), a biopolymer (Chit), a starch (Sta) and two metal salts (FeCl_3_, PACl) and concluded that cationic polymers have the most steady and best performance for fouling control. Conclusions were based on results of sludge filterability improvement and flux increase by changes in the mixed liquor characteristics, for example extracellular and soluble polymeric substances removal and particle size increase. However, the effect of flux enhancers on the biological activity in anaerobic conditions needed further study.

Iversen et al. (2008) studied the biological inhibition of different flux enhancers on aerobic sludge. Their results showed no inhibitory effects on the endogenous oxygen uptake rate with the four synthetic cationic polymers tested. However, the effect on the exogenous uptake rate was different for each polymer, namely negative, positive, and no effect. In anaerobic digestion, to the authors' best knowledge, only two reports assessing the impact of cationic polymers on the microbial activity are available. These reports showed no change on the COD removal (Díaz et al., 2014) and on the biogas production (Zhang et al., 2017) after polymer addition. However, in different research fields, polyDADMAC has been reported as an anti-microbial agent (Zhao et al., 2016; Tran et al., 2017; Wang et al., 2017) that can physically disrupt the prokaryotic cell wall. Another possible effect of a cationic polymer on anaerobic sludge is the decrease in substrate availability caused by diffusion limitation inside the formed aggregates (Kooijman et al., 2017). This is, the addition of cationic polymers causes the neutralization of charges promoting the formation of large aggregates (Gregory and Barany, 2011), consequently the surface to volume ratio decreases and a diffusion limitation inside the aggregate could be observed. Pavlostathis and Giraldo-Gomez (1991) suggested that internal mass transfer limitations are significant at aggregate sizes above 0.8 mm for acetate removal. With polymer additions we obtained aggregates with a mean diameter below 0.003 mm for all polymer concentrations used. Therefore, diffusion limitations were not considered in this research. Contradictory reports are published on the effect of cationic polymer on the microbial community. Consequently, prior to the application of a flux enhancer in an AnMBR, the possible effect on biomass activity needs to be studied.

The biochemical conversion processes of anaerobic digestion are hydrolysis, acidogenesis, acetogenesis and methanogenesis. These processes are carried out by complex microbial communities. Methanogenesis is carried out by acetoclastic and hydrogenotrophic methanogens. In a conventional mesophilic digester, the slow-growing acetoclastic methanogens are responsible for approximately 70 % of the methane produced, and are generally considered the most sensitive to the presence of inhibitors (Astals et al., 2015). Therefore, the classical approach to study the inhibitory effect of a specific compound on the biomass activity is by studying its effect on the acetoclastic methanogens.

Since models are a mathematical encoding of a more complex and detailed system or process, they do not exactly capture reality. As such, models can have both structural and parametric uncertainties. Nevertheless, models are a powerful tool to reveal insight into the processes and interactions in a given system. The most common approach to model biological inhibition in anaerobic digestion, including weak acid/base, hydrogen, pH and cation inhibition, is the biostatic inhibition that considers the effect on growth and substrate uptake kinetics, and which is included in the Anaerobic Digestion Model No. 1 (Batstone et al., 2002). Furthermore, the disruption of the cells caused by a substance is considered a reactive irreversible toxicity and it is defined as biocidal inhibition (Batstone et al., 2002). Knowing the underlying inhibition mechanisms allows understanding of the long-term implications in a continuous reactor. For example, if the polymer exerts a biocidal effect on the microorganisms, then cell lysis will likely occur resulting in a release of soluble polymeric substances. The latter compounds are reported to deteriorate the sludge filterability (Krzeminski et al., 2012), leading to the need for more polymer addition to counteract the effect. A biocidal effect is irrecoverable, therefore continuously dosing of polymers can lead to severe biomass death and the need for re-inoculation. However, if the polymer will exert biostatic inhibition, it will not have a direct impact on filterability, and the effect on the biology will immediately recover when the polymer concentration decreases in the system. Additionally, depending on the dosage of the polymer and the microbial growth rate, the overall microbial activity might be recovered by an increase in the biomass content.

In this research, we assessed the effect of a cationic polymer concentration on the biological activity of anaerobic sludge. We used modeling as a tool to study the inhibition mechanism of the polymer on the acetoclastic methanogenesis. We compared biostatic and biocidal inhibition models based on their capacity to predict the dynamic methane production in batch experiments. The models were calibrated to fit the experimental data, namely the specific methanogenic activity (SMA) and the accumulated methane production (AMP) obtained in batch tests at different concentrations of polymer. During the calibration procedure, we used the Monte Carlo (MC) method to identify the parameters subsets composed only of influential parameters and to define the boundaries of these parameters. Additionally, using the MC method we studied the uncertainty in the models' predictions caused by the uncertainty in the estimators.

## Materials and Methods

### Analytical Methods

We measured chemical oxygen demand (COD) using Hach Lange test kits, the total suspended solids (TSS) and volatile suspended solids (VSS) concentration following Standard Methods for the Examination of Water and Wastewater (American Public Health Association, 1999) and the particle size distribution by using a Microtrac Bluewave diffraction analyzer (Malvern Instruments Ltd., UK). We reported the particle size diameter as 50th percentile (*D50*), which is the diameter at which 50 % of the sample's mass is comprised of particles with a diameter less than this value. All variables were measured in triplicate, immediately before and after the batch tests experiments. Reported results are averages of the triplicates.

### Batch Reactor Tests

We assessed the effect of increasing polymer concentration on the SMA using Adifloc KD451 (Adipap, France), which is a low molecular weight cationic polymer. The polymer was selected based on its capacity to significantly enhance the sludge filterability of municipal and industrial sludge samples when applied at very low concentrations, while no effect on pH was observed (Odriozola et al., 2018).

We performed the inhibition tests in 250 mL Schott glass bottles (200 mL filled with liquid and 50 mL as head-space) under mesophilic conditions using sodium acetate as carbon source. We collected the inoculum at an anaerobic digester of a near-by sewage treatment plant (Harnaschpolder, Den Hoorn, The Netherlands). The characteristics of the inoculum were as follows: 29.3 gTSS L^−1^, 21.0 gVSS L^−1^ and *D50* of 50 nm. We pre-mixed the polymer with the inoculum, in 1 L jars of a jar-test apparatus by mixing at 90 rpm during 30 min. We filled each SMA bottle with 2.5 gCOD L^−1^ of sodium acetate, inoculum-polymer mixture, 0.6 mL L^−1^ micro and 6 mL L^−1^ macro nutrients solutions (Muñoz Sierra et al., 2018), 10 mM phosphate buffer solution at pH 7.0 (Spanjers and Vanrolleghem, 2016) and demineralized water, and then flushed the bottles with nitrogen gas for 1 min. The inoculum concentration in the bottles was 4 gVSS L^−1^ (corresponding to 6 gTSS L^−1^), and we used the following concentrations of polymer: 0, 0.06, 0.11, 0.17, 0.23, 0.28, 0.34, 0.40, and 0.46 gCOD L^−1^. The maximum concentration of polymer tested was approximately ten times the concentration of KD451 applied to a pilot AnMBR for fouling control (Odriozola et al., 2019), namely 0.05 gCOD L^−1^. We performed the SMA tests in triplicate and placed the bottles inside an orbital shaker at 130 rpm with temperature control at 35°C and over a 10-day period.

We determined the methane production using an “automated methane potential test system” (AMPTS from Bioprocess Control, Sweden). The AMPTS generates a digital pulse after a fixed volume of gas (~10 mL) has flowed through the gas cells, and measures the temperature and pressure in the water bath containing the gas cells. The AMPTS calculates and records the volume of gas under normal conditions (N-mL, 0°C, 1 bar). We calculated the AMP, expressed in kgCOD kgVSS^−1^, by dividing the data recorded in the AMPTS by the mass of VSS inoculated and by the stoichiometric methane production per kg COD, i.e., 3.5 × 10^5^ N-mL kgCOD^−1^. We calculated the SMA following Spanjers and Vanrolleghem (2016).

### Mathematical Models Description

In this research, we compared the results from five different models, predicting the methane production from acetate in batch reactors in the presence of an inhibitory compound (the polymer). With the first three models, M1a to M3a, we described the biostatic inhibition of the acetate degradation by the concentration of inhibitor in the bulk liquid. The biostatic models assume that the inhibitor binds to the enzyme or the complex enzyme-substrate and does not allow the product formation. The kinetic models considered were as follows: competitive (M1a) where the inhibitor attaches to the enzyme in the same place as the substrate, non-competitive (M2a) where the inhibitor attaches to the enzyme in a different place changing the structure of the enzyme, and un-competitive (M3a) where the inhibitor attaches to the complex enzyme-substrate (Garcia Orozco, 2008). In the fourth (M4a) and fifth (M5a) models we described the biocidal effect of the inhibitor concentration in the bulk liquid on the microbial decay. In M4a we included a linear model describing the decay rate change with the inhibitor concentration, and M5a an exponential model.

We considered the following soluble components: total acetate (*ac*), methane gas (*ch*4), inorganic carbon (*IC*), carbon dioxide gas (*co*2), nitrogen gas (*n*2), and inhibitor (polymer) (*I*); and particulate components as follows: acetate degraders and adsorbed polymer. The model included the following processes: adsorption of the polymer (inhibitor) into the biomass, uptake of acetate by methanogens, liquid-gas transfer of nitrogen, carbon dioxide and methane, acid-base equilibria for inorganic carbon and biomass decay. The uptake of acetate was assumed to be performed by the dispersed biomass in the bulk liquid.

#### Polymer Adsorption

In this research we assumed that equilibrium conditions for polymer adsorption were achieved after 30 min mixing the inoculum with the polymer, as shown by other authors for the absorption of PolyDADMAC onto waste activated sludge (Zhao et al., 2016) and onto cellulosic fibers (Horvath et al., 2006). We used the Langmuir adsorption isotherm to describe the equilibrium conditions as follows:

(1) $$\begin{array}{r} Q_{e}=Q_{m}\frac{K_{L}C_{e}}{1+K_{L}C_{e}} \end{array}$$

where *C*_*e*_ (kgCOD m^−3^) is the concentration in the bulk liquid after equilibrium, *Q*_*e*_ (kgCOD kgTSS^−1^) the adsorbent phase concentration after equilibrium, *Q*_*m*_ (kgCOD kgTSS^−1^) the maximum adsorption capacity corresponding to monolayer coverage and *K*_*L*_ (m^3^ kgCOD^−1^) the Langmuir affinity coefficient.

The mass balance of polymer inside the reactor was as follows:

(2) $$\begin{array}{r} Q_{e}=\left(C_{0}-C_{e}\right)\frac{V}{M_{R}} \end{array}$$

where *C*_0_ (kgCOD m^−3^) is the initial concentration in the bulk liquid, *V* (m^3^) the volume of liquid in the reactor and *M*_*R*_ (kgTSS) the mass of adsorbent (or total solid content) inside the reactor. Therefore, we estimated the equilibrium concentrations *C*_*e*_ and *Q*_*e*_ by combining Equation (1) and Equation (2). We determined experimentally the values of *C*_0_, *V*, and *M*_*R*_, and estimated *Q*_*m*_ and *K*_*L*_ by fitting the model to the experimental data. We assumed the concentration of polymer in the bulk liquid (*S*_*I*_, kgCOD m^−3^) as equal to the equilibrium concentrations, namely *S*_*I*_ = *C*_*e*_.

#### Kinetic Processes

We included the conversion of acetate to methane and inorganic carbon by acetoclastic methanogenic archaea, and the biomass decay processes in the kinetic models, as summarized in Table 1. In models M1a, M2a, and M3a, we considered the biostatic inhibition (competitive, non-competitive and un-competitive) of the acetate degradation rate (ρ_1_, kgCOD m^−3^ d^−1^) by the concentration of inhibitor in the bulk liquid.

**Table 1:** Description of kinetic process used in the evaluated models.

**Model**	**Inhibition type**	**Uptake of acetate (ρ_1_)^a^**	**Decay of acetate degraders (ρ_5_)^b^**
M0	No inhibition	$k_{m,ac}\frac{S_{ac}}{K_{s.ac}{+S}_{ac}}X_{ac}$	*k*_*d*_*X*_*ac*_
M1a	Biostatic, competitive	$k_{m,ac}\frac{S_{ac}}{K_{s.ac}\left(1+\frac{S_{I}}{K_{I}}\right){+S}_{ac}}X_{ac}$	*k*_*d*_*X*_*ac*_
M2a	Biostatic, non-competitive	$k_{m,ac}\frac{S_{ac}}{\left(K_{s.ac}{+S}_{ac}\right)\left(1+\frac{S_{I}}{K_{I}}\right)}X_{ac}$	*k*_*d*_*X*_*ac*_
M3a	Biostatic, un-competitive	$k_{m,ac}\frac{S_{ac}}{K_{s.ac}{+S}_{ac}\left(1+\frac{S_{I}}{K_{I}}\right)}X_{ac}$	*k*_*d*_*X*_*ac*_
M4a	Biocide, linear	$k_{m,ac}\frac{S_{ac}}{K_{s.ac}{+S}_{ac}}X_{ac}$	$\frac{S_{I}}{K_{I}}k_{d}X_{ac}$
M5a	Biocide, exponential	$k_{m,ac}\frac{S_{ac}}{K_{s.ac}{+S}_{ac}}X_{ac}$	$10^{\frac{S_{I}}{K_{I}}}k_{d}X_{ac}$

a *S_ac_ (kgCOD m^−3^) is the total acetate concentration, X_ac_ (kgCOD m^−3^) the concentration of acetate degraders, K_S, ac_ the Monod half saturation constant (kgCOD m^−3^), k_m, ac_ (d^−1^) the Monod maximum specific uptake rate and K_I_ (kgCOD m^−3^) the concentration of inhibitor giving 50% inhibition*.

b *k_d_ (d^−1^) is the first order decay rate*.

We included first order kinetics to describe the biomass decay rate (ρ_5_, kgCOD m^−3^ d^−1^) in all models. Additionally, in models M4a and M5a we described the biocidal inhibition by relating the concentration of inhibitor with the first order decay rate. Therefore, as shown in Table 1, we proposed a simple linear model between the first order decay rate and the concentration of inhibitor in the bulk liquid *S*_*I*_ in M4a, and an exponential term in M5a. The latter was analogous to the microbial inactivation kinetics by chemical compounds (Casolari, 1988).

#### Liquid-Gas Mass Transfer

We estimated the specific liquid-gas mass transfer rates for methane (ρ_2_, kgCOD m^−3^ d^−1^), carbon dioxide (ρ_3_, kmol m^−3^ d^−1^), and nitrogen (ρ_4_, kmol m^−3^ d^−1^) as follows (Batstone et al., 2002):

(3) $$\begin{array}{r} \rho_{2}=k_{L}a\left(S_{ch4}-K_{H,ch4}p_{gas,ch4}\right) \end{array}$$

(4) $$\begin{array}{r} \rho_{3}=k_{L}a\left(S_{co2}-K_{H,co2}p_{gas,co2}\right) \end{array}$$

(5) $$\begin{array}{r} \rho_{4}=k_{L}a\left(S_{n2}-K_{H,n2}p_{gas,n2}\right) \end{array}$$

where *k*_*L*_*a* is the dynamic gas–liquid transfer coefficient, *K*_*H,n*2_ (kmol m^−3^ bar^−1^), *K*_*H,co*2_ (kmol m^−3^ bar^−1^), and *K*_*ch*4_ (kgCOD m^−3^ bar^−1^) are the Henry's law coefficients, *p*_*gas,ch*4_, *p*_*gas,co*2_ and *p*_*gas,n*2_ (bar) the partial pressures of gases, and *S*_*ch*4_ (kgCOD m^−3^), *S*_*co*2_ (kmol m^−3^), and *S*_*n*2_ (kmol m^−3^) the concentrations of methane, carbon dioxide and nitrogen in the liquid phase, respectively. We assumed the same value for *k*_*L*_*a* for all gaseous components.

We estimated the composition of the gas phase assuming that gas-liquid equilibrium was reached for all gaseous components in accordance to Henry's law, and that the total gas pressure *p*_*gas*_ (bar) was the sum of the partial pressures of all the gaseous components inside the reactor. Consequently, we estimated the partial pressure *p*_*gas,i*_ as follows:

(6) $$\begin{array}{r} p_{gas,i}=p_{gas}\frac{\frac{S_{i}}{K_{H,i}}}{\frac{S_{n2}}{K_{H,n2}}+\frac{S_{ch4}}{K_{H,ch4}}+\frac{S_{co2}}{K_{H,co2}}} \end{array}$$

We used the acid-base equilibrium equation for inorganic carbon (CO_2,ac_/${\text{HCO}}_{3}^{-}$) to estimate the *S*_*co*2_ from the concentration of inorganic carbon in the liquid phase (*S*_*IC*_, kmol m^−3^), as follows:

(7) $$\begin{array}{r} S_{co_{2}}=S_{IC}\left(1-\frac{K_{a,co_{2}}}{K_{a,co_{2}}+10^{-pH}}\right) \end{array}$$

where *K*_*a, C*_*O*__2__ (-) is the acid-base equilibrium coefficient and *pH* (-) is the pH of the solution. In this research, we considered a constant pH, because we added a pH-buffer into the bottles in the batch-tests experiments.

#### Mass Balances

We estimated the AMP (*P*_*ch*4_, kgCOD kgVSS^−1^) from the specific liquid-gas mass transfer rates (ρ_2_), as follows:

(8) $$\begin{array}{r} \frac{\partial P_{ch4}}{\partial t}=\frac{\rho_{2}}{t_{c}X\left(0\right)} \end{array}$$

where *X*(0) (kgVSS m^−3^) is the initial concentration of VSS in the reactor, experimentally determined, and *t*_*c*_ (86,400 s d^−1^) is a time conversion factor.

We used the following mass balance equation for component *i* in the bulk liquid:

(9) $$\begin{array}{r} \frac{\partial C_{B,i}}{\partial t}=\frac{1}{t_{c}}\underset{j=\left[1-6\right]}{\sum }\nu_{ij}\rho_{j} \end{array}$$

where *C*_*B, i*_ is the concentration of the soluble (*S*_*i*_) or particulate (*X*_*i*_) component *i* in the bulk liquid, *t* (s) the time, ν_*ij*_ the stoichiometric coefficients of component *i* on process *j* and ρ_*j*_ (kgCOD m^−3^ d^−1^ or kmol m^−3^ d^−1^) the rate of process *j*. The ν_*ij*_ are presented in Table 2.

**Table 2:** Stoichiometric coefficients (υ_*i, j*_).

**Component** ***i*** **→**		**1**	**2**	**3**	**4**	**5**
**j**	**Process ↓**	***S*_*ac*_**	***S*_*ch*4_**	***S*_*IC*_**	***S*_*n*2_**	***X*_*ac*_**
1	Uptake of acetate	−1	1−*Y*_*ac*_^a^	$-\sum_{\text{i}\neq \text{3}}C_{ivi,1}$^b^		
2	Liquid-gas transfer of *ch*4		−1			
3	Liquid-gas transfer of *co*2			−1		
4	Liquid-gas transfer of *n*2				−1	
5	Decay of *X*_*ac*_					−1

a *Y_ac_ is the yield coefficient*.

b *C_i_ (kmole kgCOD^−1^) is the carbon content of component i*.

### Model Implementation

#### Computational Implementation

We performed all computations in Matlab® R2018a and used the built in ordinary differential equations (ODE) solver *ode15s* to integrate the ordinary differential equations system. The initial conditions and parameters of the experiments needed for model resolution were: *p*_*gas*_, *pH*, *V*, *M*_*R*_, *C*_0_, *S*_*i*_(0) and *X*_*ac*_(0). We calculated the SMA simulations (kgCOD kgVSS^−1^ d^−1^) as the maximum methane production rate, as follows:

(10) $$\begin{array}{r} \text{SMA prediction}=\text{max}\left(\frac{\Delta P_{ch4}}{\Delta t}t_{c}\right) \end{array}$$

We estimated the goodness of fit based on the sum of squared errors (SSE), as follows:

(11) $$\begin{array}{r} \text{SSE}=\sum_{\;i\;}{\left(y_{e,i}-y_{m,i}\right)}^{2} \end{array}$$

where *y*_*e,i*_ is the experimental observation *i* and *y*_*m,i*_ the corresponding model prediction. We estimated the SSE both using SMA as the output variable (SSE-SMA, kgCOD^2^ kgVSS^−2^ d^−2^) and using the AMP as output variable (SSE-AMP, kgCOD^2^ kgVSS^−2^). We used different polymer concentrations *C*_0_ in each SMA tests (see section Batch Reactor Tests); thus, we solved the models (M1a to M5a) individually for each *C*_0_ tested to estimate the SSE.

As described in section Batch Reactor Tests, the AMPTS generates a digital pulse after a fixed volume of gas has flowed through the gas cells; consequently, the time at which each data point was measured was different for each bottle, even for the triplicates of the same SMA test. Therefore, to estimate the SSE-AMP, the models needed to predict the methane production at the exact experimental time instant. To achieve this, the models were solved with a sufficiently small time step (that is *t* = 1000 s), and the AMP prediction values were linearly interpolated at the exact experimental time instants for each SMA bottle.

#### Nominal Values of Parameters

The values of most parameters were obtained from literature, and are summarized in Table 3. The values of parameters related to the polymer characteristics, namely *Q*_*m*_, *K*_*L*_, and *K*_*I*_, were selected based on specific assumptions, and the most influential ones estimated to fit the experimental data. There are several commercial cationic polymers with similar compositions but each of them with different charges and molecular weights. To our best knowledge, there are no specific values for the parameters related to the polymer characteristics reported in literature. The parameters for the Langmuir adsorption model, *Q*_*m*_ and *K*_*L*_, are conditioned to the type of adsorbent and adsorbate. We used experimental data for the adsorption of polyDADMAC onto different adsorbents from previous reports and estimated the values of *Q*_*m*_ and *K*_*L*_. The estimated values of *Q*_*m*_ were 0.032, 0.450, and 0.035 kg kg^−1^ for adsorption onto silica gel of 6 nm pore size (Hubbe et al., 2011), activated sludge (Zhao et al., 2016), and cellulosic fibers (Horvath et al., 2006), respectively, and those for *K*_*L*_ were 2.0, 7.6 and 1,960 m^3^ kg^−1^, respectively. *Q*_*m*_ and *K*_*L*_ results were highly dispersed, therefore we used the values proposed for adsorption onto activated sludge as an initial guess. We assumed the *K*_*I*_ equal to the *C*_*e*_ calculated using Equation (1) and Equation (2) by substituting for the values for *Q*_*m*_ and *K*_*L*_ from activated sludge and for the value for *C*_0_ equal to the experimental concentration of polymer at which the SMA value was 50% smaller than the SMA without polymer. Therefore, the *K*_*I*_ was set equal to 0.014 kgCOD m^−3^.

**Table 3:** Nominal parameter values at 35°C.

**Component** ****i**** **→**		**1**	**2**	**3**	**4**	**5**	**References**
**Parameter**	**Units**	**S_ac_**	**S_ch4_**	**S_IC_**	**S_n2_**	**X_ac_**	
*K*_*H, i*_	kmol m^−3^ bar^−1^^a^		0.108	0.027^b^	5.5 × 10^−4^		Sander, 2015
*K*_*a, CO*2_	× 10^7^			4.94			Batstone et al., 2002
*C*_*i*_	kmol kgCOD^−1^	0.0313	0.0156		0	0.0313	Batstone et al., 2002
*k*_*m, ac*_	d^−1^	8					Batstone et al., 2002
*K*_*s, ac*_	kgCOD m^−3^	0.15					Batstone et al., 2002
*Y*_*ac*_	–	0.05					Batstone et al., 2002
*k*_*d*_	d^−1^	0.1					Batstone et al., 2004
*k*_*L*_*a*	d^−1^		178	178^b^	178		Metcalf et al., 2002

a *K_H,i_ units for methane: kgCOD m^−3^ bar^−1^*.

b for CO_2._

#### Initial Conditions and Experimental Parameters

We estimated the total solids content inside the reactor (*M*_*R*_, kgTSS) as the initial concentration of TSS in the reactor, which was experimentally determined, times the liquid volume of the reactor (*V*). The initial concentration *C*_0_ in Equation (2) represents the concentration of polymer added to the SMA bottles. We assumed a constant total gas pressure *p*_*gas*_ equal to the mean experimentally measured pressure, which was 1.01 bar. We introduced the ratio between partial pressure and total gas pressure at time zero as an initial condition in each model and estimated the soluble components concentrations [*S*_*ch*4_(0), *S*_*IC*_(0) and *S*_*co*2_(0)] using Equations (6) and (7), assuming that the system starts at gas-liquid equilibrium. Since the bottles were initially flushed with nitrogen gas, we assumed that the initial $\frac{p_{gas,i}}{p_{gas}}$ were 0, 0 and 1 for methane, carbon dioxide and nitrogen, respectively.

We calculated the initial concentration of acetate degraders as *X*_*ac*_(0) = *f*_*Xac*,0_ *VSS*(0), where *f*_*Xac*,0_ (kgCOD kgVSS^−1^) is the initial fraction of acetate degraders of the VSS. We estimated the *f*_*Xac*,0_ value to fit the model M0 (Table 1) to the experimental AMP when no polymer was added to the SMA bottles. We did not include the adsorption model since no polymer was present in the reactor. We estimated the initial guess assuming that all the methane produced from acetate left the bottles and considering *S*_*ac*_≫*K*_*s,ac*_. Consequently, the initial guess for *f*_*Xac*,0_ was estimated as follows:

(12) $$\begin{array}{r} f_{Xac,0}=\frac{\text{SMA}}{k_{m,ac}\left(1-Y_{ac}\right)} \end{array}$$

where the SMA (kgCOD kgVSS^−1^ d^−1^) was experimentally measured.

### Models Calibration

As described in sections Nominal Values of Parameters and Initial Conditions and Experimental Parameters, we estimated the parameters *f*_*Xac*,0_, *Q*_*m*_, *K*_*L*_ and *K*_*I*_ to fit the experimental data. We used the AMP without polymer addition as the model output (*y*) to estimate *f*_*Xac*,0_, and for the remaining parameters we used SMA for different initial polymer concentrations *C*_0_ as the model output.

We carried out the model calibration in five steps, namely: (1) identification of a parameter subset (θ) containing only the influential parameters based on the standardized regression coefficients (β_*i*_) from the linear regression model built using the MC simulations; (2) definition of the boundaries for the parameters based on the behavior of the SSE with respect to uncertain model parameters; (3) parameter estimation (PE) with θ and evaluation of the quality of the estimators; (4) (when needed) identification of the θ that can be reliably estimated from the given experimental data, by modification of the model structure and/or identifiability analysis; and (5) PE with new θ and/or model. Additionally, we performed a model prediction uncertainty analysis using the MC method with the parameter uncertainty obtained from PE.

#### Monte Carlo and Linear Regression

We performed a global sensitivity analysis to identify the effect of the parameters on the model output. We executed the analysis based on linear regression models built from MC simulations. We defined the input uncertainty as a uniform distribution with 99.9 % variability, we used 99.9 % instead of 100 % to avoid null values; zero values for some parameters would cause numerical problems, for example, if *K*_*H,i*_ is zero (for any *i*) then there is a division by zero in Equation (6). Consequently, the minimum and maximum value of the distributions were 0.001θ° and 1.999θ°, respectively, where θ° is the initial/nominal parameter vector. The notation θ ~ *U*(0.001θ°, 1.999θ°) is further used in this document. The θ included all parameters in each model. We used Latin hypercube sampling (Iman and Conover, 1982) with 500 samples (Sin et al., 2009); computed the β_*i*_ using the mean-centered sigma-scaling (Helton and Davis, 2003), and estimated the β_*i*_ using the constrained linear least square minimization function *lsqlin* with −1 and 1 as lower and upper bound, respectively. The estimation of the β_*i*_ requires a scalar input; therefore we computed the β_*i*_ individually for each *C*_0_ in M1a to M5a and for each time (*t*) in M0. We used the coefficient of determination (*R*^2^) to evaluate the quality of the regression model, that is: the model was considered sufficiently linear when *R*^2^ ≥ 0.7 (Sin et al., 2011). We calculated the mean, minimum and maximum *R*^2^ with the β_*i*_ obtained for each parameter in the range of *C*_0_ or *t* were *R*^2^ ≥ 0.7. The parameters with a mean abs(β_*i*_) ≥ 0.10 were considered influential (Sin et al., 2011).

#### Parameter Estimation

We studied the behavior of the SSE with the parameter values in the subset θ varying in a wide range to research the existence of local minimums and determine the boundaries and initial guess for the PE. We performed MC simulations with Latin hypercube sampling with 500 samples, using a uniform distribution with 99.9% variability for the parameter subset defined in section Monte Carlo and Linear Regression and calculated the SSE for each simulation. We defined the range of the parameters based on the behavior of the SSE with respect to uncertain model parameters.

Subsequently, we estimated the parameters using the non-linear least squares solver *lsqnonlin* in Matlab® R2018a, using the trust region reflective algorithm and with the lower and upper bounds previously identified. The input function for the *lsqnonlin* solver was an array with the residuals, where *Residuals* = *y*_*e*_−*y*_*m*_. Afterwards, the solver found the optimal value ($\hat{\theta }$) that minimizes the sum of squares of the input function, consequently, it minimizes the SSE in Equation (11). We calculated the standard deviation (σ_θ_) and 95 % confidence interval (CI) of the estimators in accordance to Sin and Gernaey (2016).

The normality of the residuals needs to be evaluated since it is an underlying hypothesis for the implementation of least square method for parameter estimation. We assessed the distribution of the residuals graphically and used Shapiro-Wilk test to test the hypothesis of normality using the function *swtest*© (BenSaïda, 2009). When the null hypothesis of normality was rejected at a significance level 0.05, we estimated the parameters using bootstrap method, implemented as described in Sin and Gernaey (2016).

We assessed the quality of the estimators based on the uncertainty of the estimators and the pairwise linear correlation between the parameters and considered a good estimation when the relative error, namely $\frac{\sigma_{\theta }}{\hat{\theta }}$, was below 10 % and a poor estimation when it was above 50 % (Sin and Gernaey, 2016). Additionally, if the correlation coefficient between any pair of parameters was above 0.5, we assumed that the PE problem was ill conditioned. Then, we performed an identifiability analysis to select the parameter subset that can be identified uniquely from the experimental data. Therefore, we computed the collinearity index of the parameter subset *k* (γ^*k*^) (Sin and Gernaey, 2016). The threshold to select a uniquely parameter subset is reported between 5 and 15 (Sin and Gernaey, 2016). We selected the parameter subset with γ^*k*^ below 10 for the PE.

### Modeling Scenarios

Two different scenarios were studied. The first scenario (models a: M1a, M2a, M3a, M4a, and M5a), considered that the inhibition is caused by the polymer (inhibitor) present in the bulk liquid. As described in section Polymer Adsorption, when a polymer is added to a sludge sample a fraction of the polymer is adsorbed onto the sludge and a fraction remains in the bulk liquid. The concentration of polymer in the bulk liquid after the adsorption reaching equilibrium is *C*_*e*_, and *C*_*e*_ was considered as the concentration of polymer responsible for the inhibition in the first scenario, namely *S*_*I*_ = *C*_*e*_.

In the second scenario (models b: M1b, M2b, M3b, M4b, and M5b), the inhibition was assumed to be caused by the total amount of polymer added to the SMA bottles. The inhibition is caused by both the fraction of polymer adsorbed onto the sludge and the fraction remaining in the bulk liquid. Consequently, the adsorption model was not needed to describe the inhibition and it was removed from the model structure in models b and the *S*_*I*_ was set equal to the total concentration of polymer added to the SMA bottles (*C*_0_), namely *S*_*I*_ = *C*_0_.

### Model Prediction Uncertainty

We selected the most suitable models based on the model capacity to predict the experimental data (goodness of fit), which was assessed graphically and with the SSE-SMA and SSE-AMP. For the selected models, we studied the model prediction uncertainty caused by the uncertainty in the estimators, using the MC method with Latin hypercube sampling. We defined the uncertainty in the parameters using the results from the PE, as a normal distribution with mean $\hat{\theta }$ and standard deviation σ_θ_, namely $\theta \sim N\left(\hat{\theta },{\sigma_{\theta }}^{2}\right)$, and represented the uncertainty propagation graphically.

## Results

### Experimental Results

The polymer presented an inhibitory effect on the SMA. The SMA of the sludge without polymer was 0.219 gCOD gVSS^−1^ d^−1^. A 50 % SMA inhibition was obtained at 0.27 gCOD L^−1^ of polymer, the value was obtained by linear interpolation (see Supplementary Material). No significant effect was observed in the AMP achieved at the end of the tests, for further information see the Supplementary Material.

### Modeling AMP Without Polymer Addition (M0)

**Figure 1:**
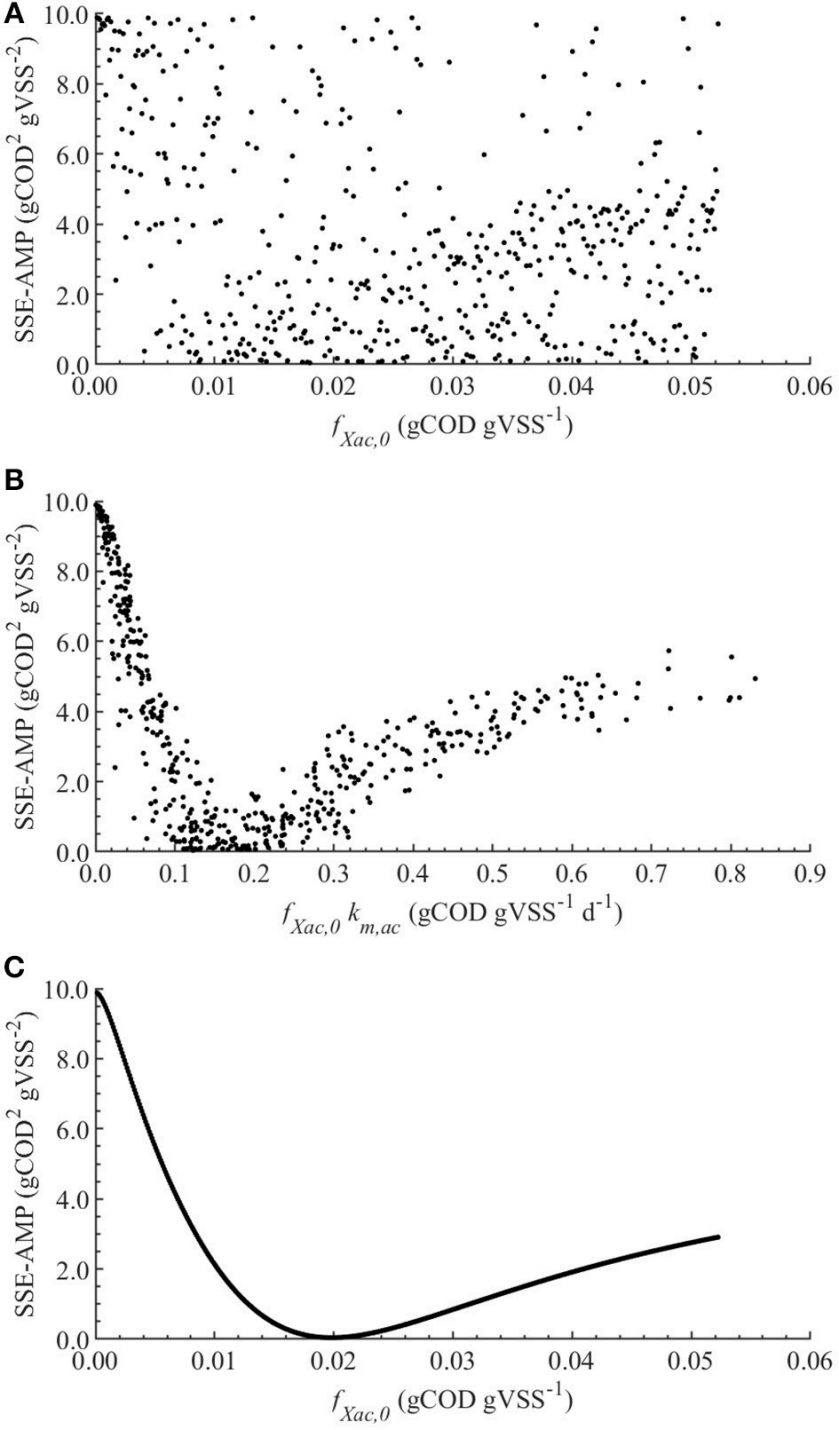
Correlations between the SSE-AMP and uncertain parameters with θ ~ *U*(0.001θ°, 1.999θ°) for M0: correlation of SSE-AMP with *f*_*Xac*,0_ **(A)** and SSE-AMP with *f*_*Xac*,0_*k*_*m,ac*_ **(B)** using MC simulations performed with uncertainty in all parameters; correlation of SSE-AMP with *f*_*Xac*,0_ using MC simulations performed with uncertainty only in the *f*_*Xac*,0_ **(C)**.

The linear regression models built using the MC simulations of M0 resulted in *R*^2^ above 0.7 between 2 and 79 h. The mean, minimum and maximum β_*i*_ values for the range with *R*^2^ > 0.70 are presented in Table 4. The SSE-AMP was calculated for each MC simulation and the results are presented in Figure 1. The behavior of the SSE-AMP with *f*_*Xac*,0_, for uncertainty only in *f*_*Xac*,0_, is shown in Figure 1C.

**Table 4:** Sensitivity analysis results for M0: mean, minimum (min) and maximum (max) of the standardized regression coefficients (β_*i*_) of linear models with *R*^2^ > 0.7 of AMP as a function of time.

**Parameter**	**M0 (mean** ****R****^****2****^ **=** **0.81)**		
	**Mean**	**Min**	**Max**
*f*_*Xac*,0_	0.52	0.43	0.60
*k*_*m, ac*_	0.71	0.56	0.75
*K*_*s, ac*_	−0.05	−0.06	0.01
*Y*_*ac*_	0.05	−0.05	0.07
*k*_*d*_	−0.03	−0.05	−0.02
*k*_*L*_*a*	0.03	0.02	0.10
*K*_*H,ch*4_	−0.09	−0.30	−0.07
*K*_*H,co*2_	0.00	−0.02	0.02
*K*_*H,n*2_	0.03	0.02	0.04
*K*_*a, C*_*O*__2__	0.02	−0.04	0.06
*C*_*Xac*_	−0.01	−0.02	0.01

**Figure 2:**
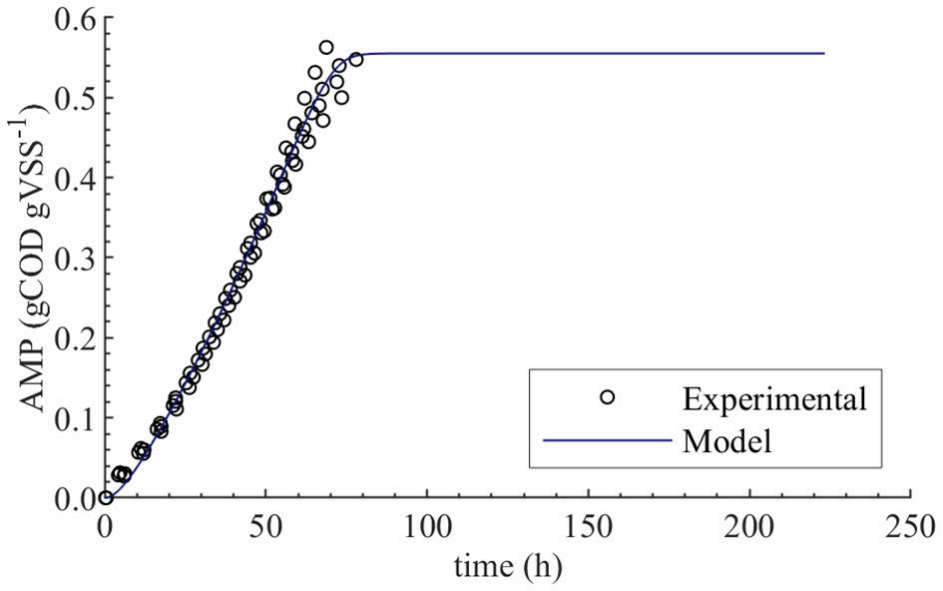
Experimental and simulated AMP in the absence of polymer (M0). Simulations performed with the optimum value obtained from parameter estimation, namely *f*_*Xac*,0_ = 0.0198 kgCOD kgVSS^−1^.

The initial guess for *f*_*Xac*,0_ estimation was 0.02, and the lower and upper bounds were 0.01 and 0.03, respectively. The PE results were as follows: 0.0198 kgCOD kgVSS^−1^ optimal value ($\hat{\theta }$), 1.9 × 10^−4^ standard deviation (σ_θ_), 3.8 × 10^−4^ 95 % confidence interval (CI) and relative error $\frac{\sigma_{\theta }}{\hat{\theta }}$ of 1 %. We performed PE varying the initial guess and the same results were obtained. The null hypothesis of normality was rejected at a significance level 0.05 using the Shapiro-Wilky test with a *p-*value of 2 × 10^−7^. The optimal value obtained using Bootstrap was equal to the one obtained with least squares method, namely 0.0198 kgCOD kgVSS^−1^. Figure 2 displays the experimental AMP and the model predictions at the optimal values obtained with PE. Although the experimental data was collected over a 10-day period, not enough biogas to generate a new pulse in the AMPTS was produced in the bottles after 80 h, further explanation was presented in Supplementary Material.

### Modeling the Methanogenesis Inhibition by the Polymer

The results from the linear models performed with the MC simulations with parameter uncertainty θ ~ *U*(0.001θ°, 1.999θ°) are summarized in Table 5. From the parameters present in the initial subsets θ, the ones with mean β_*i*_ ≥ 0.10 were: {*K*_*I*_, *K*_*L*_, *Q*_*m*_} in M1a to M4a and {*K*_*I*_, *K*_*L*_} in M5a.

**Table 5:** Sensitivity analysis results for M1a to M5a: mean of the standardized regression coefficients (β_*i*_) and mean *R*^2^ ($\bar{R^{2}}$) of linear models with *R*^2^ > 0.7 of the SMA with different concentrations of polymer.

**Parameter↓ $\bar{R^{2}}$→**	**M1a**	**M2a**	**M3a**	**M4a**	**M5a**
	**0.80**	**0.80**	**0.80**	**0.83**	**0.80**
*K*_*I*_	0.17	0.19	0.20	0.18	0.15
*K*_*L*_	0.12	0.15	0.17	0.11	0.10
*Q*_*m*_	0.13	0.17	0.20	0.16	0.08
*k*_*m, ac*_	0.76	0.78	0.69	0.77	0.79
*K*_*s, ac*_	−0.24	−0.14	−0.14	−0.15	−0.13
*Y*_*ac*_	0.28	0.30	0.30	0.31	0.32
*k*_*d*_	−0.09	−0.11	−0.08	−0.12	−0.12
*k*_*L*_*a*	0.02	0.03	0.00	−0.02	−0.01
*K*_*H,ch*4_	−0.01	0.00	−0.01	−0.05	−0.02
*K*_*H,co*2_	0.01	−0.01	0.00	−0.01	0.01
*K*_*H,n*2_	−0.01	0.01	−0.02	−0.03	0.02
*K*_*a, C*_*O*__2__	0.00	−0.01	0.05	−0.01	−0.02
*C*_*Xac*_	0.03	0.02	0.01	0.01	0.00

**Figure 3:**
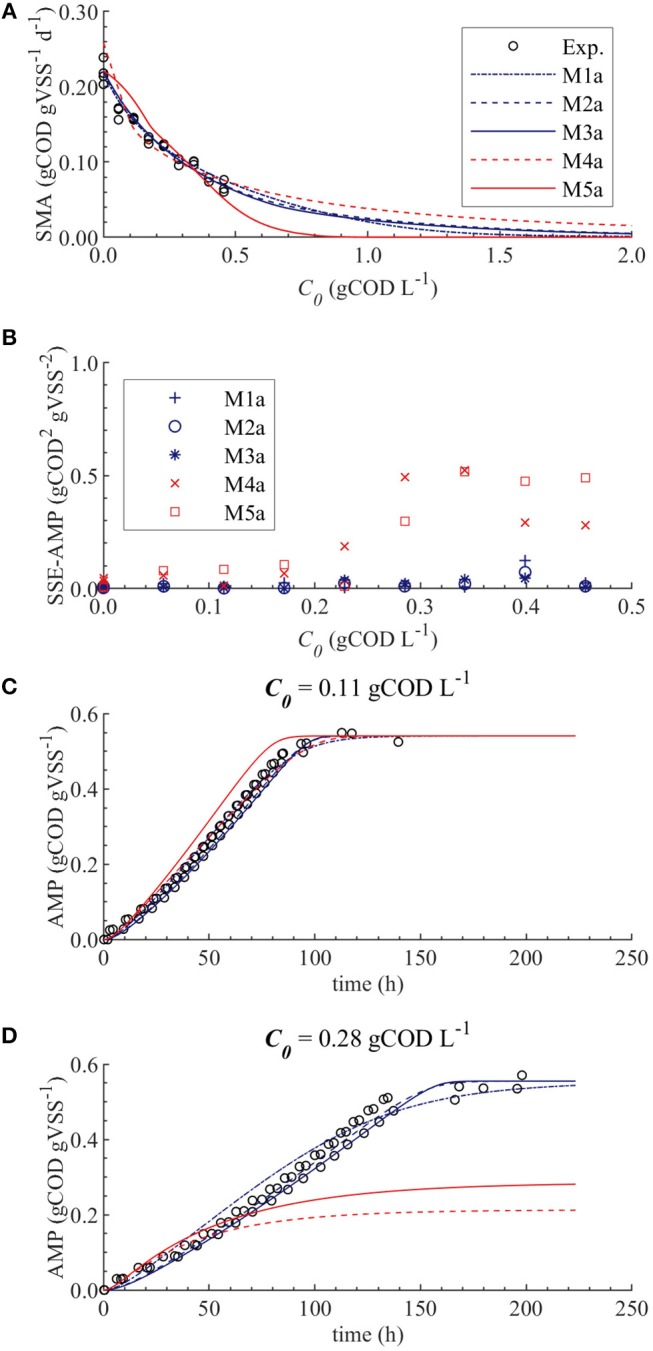
Simulations with parameter estimation results for the SMA inhibition for M1a to M4a with parameter subset θ = {*K*_*I*_, *K*_*L*_, *Q*_*m*_} and M5a with parameter subset θ = {*K*_*I*_, *K*_*L*_}: experimental and simulated SMA **(A)**, SSE-AMP **(B)**, experimental and simulated AMP at 0.11 gCOD L^−1^ of polymer **(C)** and experimental and simulated AMP at 0.28 gCOD L^−1^ **(D)**.

The results from the PE in M1a to M5a are shown in Table 6. Figure 3 shows the comparison between the best-fit results of the five models and the experimental data. The correlation coefficients and collinearity index for all the possible combinations of parameters are summarized in Table 7. Based on the results, a new parameter subset was defined containing only *K*_*I*_, and the values of *K*_*L*_ and *Q*_*m*_ were set at their nominal values (defined in section Nominal values of parameters)

**Table 6:** Parameter estimation results for the SMA inhibition for M1a to M4a with parameter subset θ = {*K*_*I*_, *K*_*L*_, *Q*_*m*_} and M5a with θ = {*K*_*I*_, *K*_*L*_}: optimal values ($\hat{\theta }$), standard deviation (σ_θ_) and 95% confidence intervals (*CI*); and sum of square errors for SMA (SSE-SMA) and AMP (SSE-AMP) of the models.

**Parameter**	**Model**	$\hat{\theta }$	**σ_θ_**	**95% CI**	$\frac{\sigma \theta }{\hat{\theta }}\times 100$	**SSE-SMA**	**SSE-AMP**
*K*_*I*_	1	0.001	0.029	0.060	2529 %	0.0021	0.67
	2	0.014	0.031	0.065	231 %	0.0027	0.48
	3	0.014	0.021	0.044	152 %	0.0027	0.57
	4	0.005	0.076	0.158	1400 %	0.0122	7.26
	5	0.016	0.112	0.231	689 %	0.0101	7.81
*K*_*L*_	1	11.1	12.2	24.0	110 %		
	2	152.6	1025.6	2014.9	672 %		
	3	61.9	383.6	753.7	620 %		
	4	2.0	4.2	8.3	211 %		
	5	6.4	22.6	44.4	351 %		
*Q*_*m*_	1	0.17	0.05	0.10	29 %		
	2	0.24	0.03	0.06	13 %		
	3	0.24	0.04	0.08	18 %		
	4	0.45	0.47	0.92	104 %		

**Table 7:** Correlation and collinearity index for M1a to M5a for different parameter combinations *k*.

**Model↓ k →**	**Collinearity index (**γ****^******k******^**)**				**Correlation coefficient**		
	**{K_I_**,** K_L_}**	**{K_I_**,** Q_m_}**	**{K_L_**,** Q_m_}**	**{K_I_**,** K_L_**,** Q_m_}**	**{K_I_**,** K_L_}**	**{K_I_**,** Q_m_}**	**{K_L_**,** Q_m_}**
1	93	15	13	1828	−0.9999	−0.99	0.98
2	135	18	16	2143	−0.92	0.35	−0.70
3	134	19	16	2139	−0.74	−0.98	0.60
4	116	19	16	2131	−0.98	−0.94	0.87
5	283	36	32	7631	−0.99	−0.64	0.55

**Figure 4:**
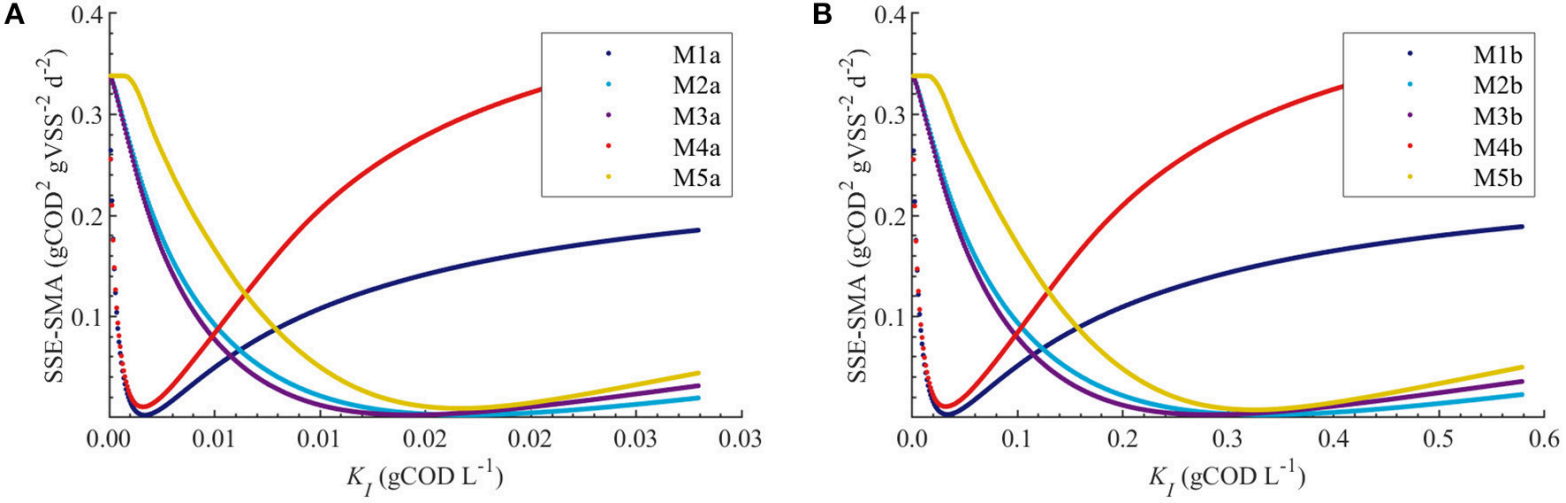
Correlations between the SSE-SMA and uncertain parameters using MC simulations with uncertainty θ ~ *U*(0.001θ°, 1.999θ°) only in *K*_*I*_. Results using M1a to M5a **(A)** and M1b to M5b **(B)**.

**Figure 5:**
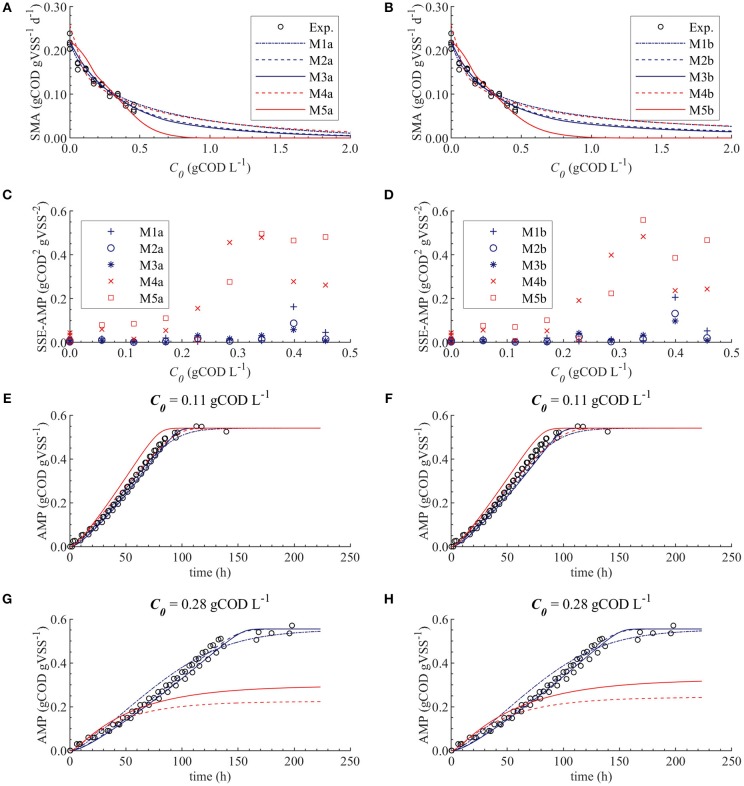
Simulations with parameter estimation results for the SMA inhibition, with parameter subset θ = {*K*_*I*_}: experimental and simulated SMA for M1a to M5a **(A)** and M1b to M5b **(B)**, SSE-AMP for M1a to M5a **(C)** and M1b to M5b **(D)**, experimental and simulated AMP at 0.11 gCOD L^−1^ of polymer for M1a to M5a **(E)** and M1b to M5b **(F)** and experimental and simulated AMP at 0.28 gCOD L^−1^ for M1a to M5a **(G)** and M1b to M5b **(H)**.

Figures 4A,B display the behavior of the SSE for SMA with $K_{I}\sim U\left(0.001\theta {}^{\circ},1.999\theta {}^{\circ}\right)$ for models a (M1a, M2a, M3a, M4a, and M5a) and b (M1b, M2b, M3b, M4b, and M5b), respectively. The results from the PE estimation are summarized in Table 8. Figure 5 compare between the best-fit results of the models with the experimental data.

**Table 8:** Parameter estimation results for the SMA inhibition process with parameter subset θ for M1a to M5a (with *S*_*I*_ = *C*_*e*_) and M1b to M5b (with *S*_*I*_ = *C*_0_).

**Model**	****θ****	$\hat{\theta }$	**σ_θ_**	**95% CI**	$\frac{\sigma \theta }{\hat{\theta }}\times 100$	**SSE-SMA**	**SSE-AMP**
1	*K*_*I*_	0.0017	4.4 × 10^−5^	0.0001	2.6 %	0.0024	0.73
1b	*K*_*I*_	0.0338	9.8 × 10^−4^	0.0019	2.9 %	0.0030	0.83
2	*K*_*I*_	0.0168	3.6 × 10^−4^	0.0007	2.2 %	0.0025	0.53
2b	*K*_*I*_	0.3341	7.5 × 10^−3^	0.0149	2.2 %	0.0028	0.63
3	*K*_*I*_	0.0143	3.1 × 10^−4^	0.0006	2.1 %	0.0026	0.61
3b	*K*_*I*_	0.2840	6.1 × 10^−3^	0.0122	2.2 %	0.0028	0.69
4	*K*_*I*_	0.0016	7.3 × 10^−5^	0.0001	4.5 %	0.0106	6.91
4b	*K*_*I*_	0.0321	1.5 × 10^−3^	0.0030	4.7 %	0.0109	6.60
5	*K*_*I*_	0.0165	4.4 × 10^−4^	0.0009	2.7 %	0.0090	7.69
5b	*K*_*I*_	0.3234	7.9 × 10^−3^	0.0158	2.4 %	0.0076	7.36

### Model Prediction Uncertainty

**Figure 6:**
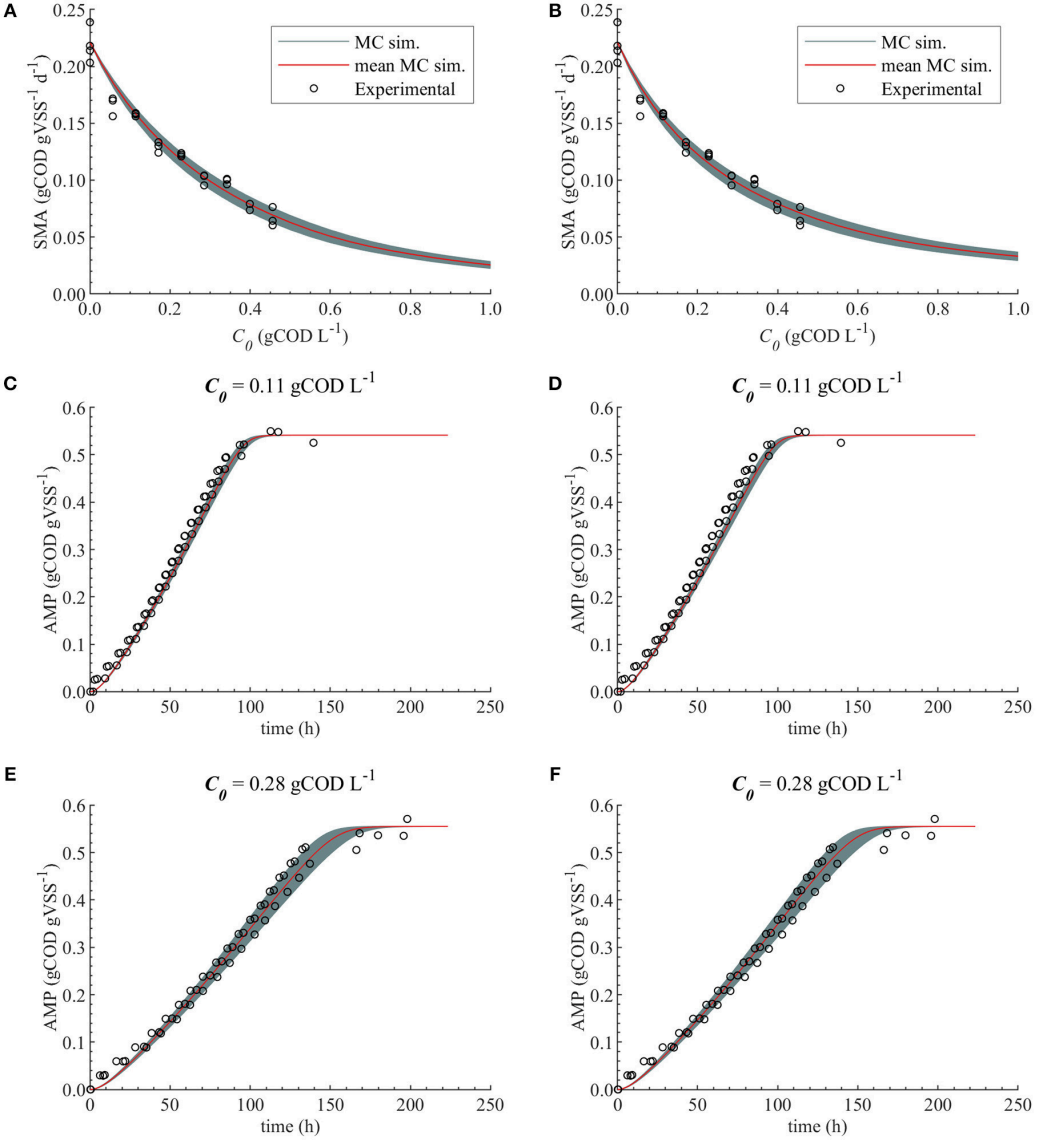
Monte Carlo simulations with uncertainty $\theta \sim N\left(\hat{\theta },{\sigma_{\theta }}^{2}\right)$ and subset θ = {*K*_*I*_} for M2a and M2b. Experimental data, MC simulations (MC sim.) and mean MC simulations for: SMA for M2a **(A)** and M2b **(B)**, AMP at 0.11 gCOD L^−1^ of polymer for M2a **(C)** and M2b **(D)** and AMP at 0.28 gCOD L^−1^ for M2a **(E)** and M2b **(F)**.

The uncertainty in the M2a and M2b predictions caused by the uncertainty in *K*_*I*_, with $\theta \sim N\left(\hat{\theta },{\sigma_{\theta }}^{2}\right)$, are compared with the experimental data in Figure 6.

## Discussion

In this research, we assessed the inhibition of SMA and AMP caused by cationic polymer addition to anaerobic sludge. The results showed that the polymer inhibited the SMA but did not have an effect on the final AMP achieved. The experimental results are further presented in the Supplementary Material.

To the authors best knowledge, two reports are available that use Adipap polymers for fouling control in membrane bioreactors. Significant fouling decrease was achieved by adding 0.05 gCOD L^−1^ of KD451 to a pilot AnMBR (Odriozola et al., 2019) and 70 mg L^−1^ (0.077 gCOD L^−1^) of KD452 to a pilot MBR (Iversen et al., 2009). These concentrations are considerably below the 50 % SMA inhibition concentration, namely 0.27 gCOD L^−1^. However, we obtained 24 and 27 % SMA inhibitions at 0.06 and 0.11 gCOD L^−1^ of polymer concentration, respectively. Consequently, in a continuous AnMBR reactor, adding polymer might decrease the methane production rate, increase the acetate concentration and decrease the organic matter removal when organic loading rates remain at the same level. The effect would start immediately after addition and it can be compensated by a decrease in the organic volumetric loading rate. Additionally, if the inhibition is reversible (biostatic) the overall conversion capacity of the bioreactor could be recovered by an increase in the biomass content. If the methanogenic microorganisms are in excess when adding the polymer to the AnMBR then the methane production, acetate concentration and organic matter removal might not be affected by small dosages of polymer.

### Validity of Nominal Values of Parameters

The results from the global sensitivity analysis showed that a 99.9% variability on the gas-liquid transfer (*k*_*L*_*a*, *K*_*H,i*_) and carbon content (*C*_*Xac*_) parameters had a negligible effect (β_*i*_ < 0.10) on the SMA inhibition (Table 5) and on the AMP without polymer (Table 4). Consequently, for those parameters, the selection of the exact (true) parameter values was not crucial and we considered it acceptable to use the nominal parameter values from literature presented in section Nominal Values of Parameters.

Contrarily, the kinetic and stoichiometric parameters related to acetate degradation and biomass decay presented a significant effect on the model output. Particularly, the Monod maximum specific uptake rate *k*_*m,ac*_ was the most influential parameter in all considered models. The *f*_*Xac*,0_ was highly correlated with *k*_*m,ac*_, meaning that the optimal value for *f*_*Xac*,0_ is determined by the value of *k*_*m,ac*_ used (Figure 1B). Although, good model fits could be obtained with different combinations of *f*_*Xac*, 0_ and *k*_*m, ac*_, this was outside the scope of this research. In this manuscript, we focused on modeling the SMA inhibition and comparing different inhibition models and not on the acetoclastic methanogenesis kinetics itself. Therefore, we used widely applied values for *k*_*m,ac*_, *Y*_*ac*_, *K*_*s,ac*_ and *k*_*d*_, presented in section Nominal Values of Parameters.

### Modeling AMP Without Polymer Addition (M0)

The initial fraction of acetate degraders of the VSS (*f*_*Xac*,0_) showed a significant effect on the AMP model output (Table 4): 28 % (${\beta_{i}}^{2}\times 100$) of the output variability could be explained by the variability on *f*_*Xac*,0_. Therefore, we could estimate the *f*_*Xac*,0_ to predict the methane production obtained experimentally. Results did not show a clear pattern of the behavior of SSE-AMP with *f*_*Xac*,0_ with uncertainty in all parameters (Figure 1A). However, the SSE-AMP with *f*_*Xac*,0_ with uncertainty only in *f*_*Xac*,0_ (Figure 1C) showed a unique (global) minimum value at *f*_*Xac*,0_ around 0.02 gCOD gVSS^−1^. Parameter estimation results showed that the quality of the estimator is good since the relative error was small and the model predictions fit the experimental data, as shown in Figure 2.

### Calibration of SMA Inhibition Models

#### Ill Conditioned Models

We identified the influential unknown parameters, for PE, using the results from the sensitivity analysis (Table 5). The subsets identified, with a threshold value of the mean β_*i*_ ≥ 0.10, are as follows: θ = {*Q*_*m*_, *K*_*L*_, *K*_*I*_} in M1a to M4a and θ = {*K*_*L*_, *K*_*I*_} in M5a.

The SMA simulations, at the optimal values obtained with PE, showed a good fit to the experimental data for the biostatic models (M1a, M2a and M3a) and the biocide-lineal model (M4a), as observed in Figure 3A. Accordingly, Figure 3C shows a good fit to the experimental AMP with M1a to M4a for a polymer concentration of 0.11 gCOD L^−1^. However, for higher concentrations (Figure 3D) the M1a simulations slightly deviated from the experimental data, and the M4a simulations were considerably below the experimental data after 50 h. The previous deviations are reflected by the SSE-AMP in Figure 3B. The accelerated biomass decay predicted in M4a caused a noticeably low concentration of microorganisms resulting in an extremely low methane production rate after 50 h. Similarly, in M5a the AMP was underpredicted for high concentrations of polymer. Additionally, the biocide-exponential model (M5a) overpredicted and underpredicted the SMA at polymer concentrations *C*_0_ below and above 0.3 gCOD L^−1^, respectively.

Models a (M1a to M5a) considered the inhibition caused by the concentration of inhibitor in the bulk liquid *S*_*I*_, where *S*_*I*_ was determined by the Langmuir isotherm adsorption model (section Polymer Adsorption). Consequently, as expected, high pairwise correlation coefficients were obtained for all parameter combinations (Table 7). As a result, the relative errors obtained with PE were considerably high (Table 6). Therefore, although some of the models were able to predict the experimental data, the quality of the estimators was considered poor, namely the relative error was above 50 % (Sin and Gernaey, 2016).

From the collinearity indexes presented in Table 7, no combination of parameters could be used to achieve unique estimators from the experimental data, namely γ^*k*^ > 10 for all *k*. Therefore, we performed a new PE with a subset containing only one parameter. We selected the inhibition coefficient *K*_*I*_ for the PE because it presented a slightly higher effect on the simulated SMA (higher mean β_*i*_, Table 5) compared to *Q*_*m*_ and *K*_*L*_, and we used the nominal *Q*_*m*_ and *K*_*L*_ values. The simulation results with the optimal values obtained with PE (Figure 5, on the left) were very similar to the ones obtained at the optimal values from PE using θ = {*Q*_*m*_, *K*_*L*_, *K*_*I*_} (or θ = {*K*_*L*_, *K*_*I*_} in M5a), Figure 3.

Additionally, we considered a different inhibition approach, where the inhibition was assumed to be caused by the total amount of polymer added to the SMA bottles, namely *S*_*I*_ = *C*_0_. Therefore, we defined the models b (M1b to M5b) and compared them with the original models a (M1a to M5a), where *S*_*I*_ = *C*_*e*_. In Figure 5 the simulation results for models a and b are displayed on the left and right graphs, respectively; the results are further discussed in section Bulk Liquid vs. Total Polymer Concentration Inhibition.

#### Biostatic and Biocidal Inhibition Models

As we discussed in section Ill Conditioned Models, biocide models (M4a and M5a) underpredicted the experimental methane production at high polymer concentrations due to the accelerated biomass decay. Therefore, the biocide models M4a and M5a were not appropriate models to describe the inhibition of methanogenesis by the polymer. Contrarily, predictions with biostatic models (M1a, M2a, and M3a) showed satisfactory fit to the experimental SMA and AMP (Figure 5). Therefore, it is likely that the polymer inhibition on the SMA is a reversible process (biostatic inhibition), instead of a reactive irreversible toxicity (biocidal inhibition). Consequently, in a continuous reactor, the inhibitory effect will be eliminated when the polymer concentration decreases. Additionally, based on the dosage of the polymer and the microbial growth rate, the overall microbial activity could be recovered by an increase in the biomass content.

Figure 5A shows that the simulations using the un-competitive (M3) and non-competitive (M2) inhibition models successfully fitted all the experimental SMA, while the competitive inhibition model (M1a) simulations slightly deviate from the SMA at the higher concentrations of polymer tested, namely 0.40 and 0.46 gCOD L^−1^. Additionally, Figure 5G shows that the M5a overpredicted the AMP between 40 and 100 h at 0.28 gCOD L^−1^, while the M2a and M3a model simulations fitted the AMP remarkably well.

Therefore, although the M1a fitted the experimental data significantly well, the process was better described by M2a and M3a. Additionally, as the competitive inhibition model (M1) considers that the inhibitor binds to the same place as the substrate (Garcia Orozco, 2008), and because the polymer (inhibitor) and the acetate (substrate) are different molecules, the latter result was not unexpected.

The difference between the un-competitive and non-competitive models could only be observed in the AMP predictions at high concentration of polymer (Figure 5G). Based on the SSE presented in Table 8 the M2a seems to predict slightly better the experimental data. However, the difference was not considered sufficient to select one model over the other and both models were considered appropriate to describe the methanogenesis inhibition process.

#### Bulk Liquid vs. Total Polymer Concentration Inhibition

The behavior of the SSE-SMA with *K*_*I*_ revealed a unique (global) minimum for all models. Figure 4 displays the SSE-SMA as a function of *K*_*I*_ for models with inhibition by the concentrations of polymer in the bulk liquid (Figure 4A) and by the total amount of polymer added to the system (Figure 4B). Results presented a similar behavior for both inhibition models, however, the *K*_*I*_ values that minimize the SSE-SMA are 20 times larger for the models with *S*_*I*_ = *C*_0_, which corresponds to the ratio between the *C*_0_ and *C*_*e*_ obtained by applying the adsorption model (section Polymer Adsorption) with the nominal parameter values and experimental conditions.

Parameter estimation results showed that the models a (with *S*_*I*_ = *C*_*e*_) presented a slightly smaller SSE-SMA and SSE-AMP for each biostatic inhibition model considered with respect to the models b (with *S*_*I*_ = *C*_0_), as shown in Table 8. However, the difference in the simulations with the models a (left plots) and models b (right plots) was negligible for each kinetic inhibition model used, as observed in Figure 5. The similarity between models a and b was due to the approximately linear relationship between *C*_0_ and *C*_*e*_ obtained using the polymer adsorption model (results not shown). This approximately linear behavior was obtained using the experimental conditions tested (*C*_0_, *M*_*R*_, and *V*) and with the Langmuir parameter values obtained by parameter estimation (*K*_*L*_ and *Q*_*m*_).

Therefore, both modeling approaches (bulk liquid or total polymer concentration inhibition) were considered appropriate to describe the methanogenesis inhibition caused by the polymer in the range of concentrations studied. Additionally, the uncertainty in the estimated parameters did not cause a considerable uncertainty on the model prediction for M2a and M2b, Figure 6.

## Conclusion

The cationic polymer showed a negative effect on the biological activity of the anaerobic sludge. A 50 % SMA inhibition was obtained at 0.27 gCOD L^−1^ of polymer whereas no significant effect on the final AMP was observed.

Different models were presented and calibrated to fit the experimental data. The Monte Carlo method was successfully applied to study the sensitivity of the model outputs to the parameters and identify the parameter subsets for parameter estimation. The collinearity indexes and pairwise correlation coefficients showed that the parameters *Q*_*m*_, *K*_*L*_ and *K*_*I*_ are all highly correlated. Based on the Monte Carlo results and collinearity indexes the parameter subsets selected for parameter estimation was θ = {*K*_*I*_} for all the models considered.

We studied an alternative modeling approach, models M1b to M5b, considering that the inhibition was caused by the total amount of polymer added to the reactor (*S*_*I*_ = *C*_0_), and not by the concentration that remains in the bulk liquid after adsorption (*S*_*I*_ = *C*_*e*_, M1a to M5a). The difference in the models simulations with both approaches, namely *S*_*I*_ = *C*_*e*_ and *S*_*I*_ = *C*_0_, was negligible for each kinetic inhibition model used.

The simulated AMP values obtained with the biocide models, namely M4a and M5a, were below the experimental AMP at high concentrations of polymer, which was caused by a rapid decay of the acetate degraders simulated. The only models that adequately fitted the experimental SMA and AMP were the non-competitive (M2a and M2b) and un-competitive (M3a and M3b) inhibition models. Therefore, it is likely that the polymer inhibition on the SMA is reversible, instead of toxic and irreversible.

The concentrations of polymer (inhibitor) in the bulk liquid giving 50 % inhibition were 0.014 and 0.017 gCOD L^−1^ for M2a and M3a, respectively; and the total concentrations of inhibitor in the reactor giving 50 % inhibition were 0.334 and 0.284 gCOD L^−1^ for M2b and M3b, respectively.

The simulated SMA obtained with M1a and M4a adequately fitted the experimental SMA. However, the simulated AMP was below the experimental AMP for those models. Therefore, it is crucial to analyze both outputs, SMA and AMP, during model calibration.

## Author Contributions

MO designed and performed the experiments. MO, EA, and HS derived the model and MO performed the computational implementation. MO, EA, HS, ML-F and JvL provided critical feedback and helped shape the research, analysis and manuscript.

### Conflict of Interest Statement

The authors declare that the research was conducted in the absence of any commercial or financial relationships that could be construed as a potential conflict of interest.

## Nomenclature

**Table:**

COD	chemical oxygen demand
MC	Monte Carlo
PE	Parameter estimation
AMP	accumulated methane production
SMA	specific methanogenic activity
SSE	sum of squared errors
VSS	volatile suspended solids
TSS	total suspended solids
**Variables and parameters**	
*C*_0_	initial concentration of polymer in the bulk liquid (kgCOD m^−3^)
*C*_*e*_	concentration of polymer in the bulk liquid after equilibrium (kgCOD m^−3^)
*C*_*i*_	carbon content of component *i* (kmole kgCOD^−1^)
*f*_*Xac*,0_	initial fraction of acetate degraders of the VSS (kgCOD kgVSS^−1^)
*K*_*a, C*_*O*__2__	acid-base equilibrium coefficient (CO_2, ac_/${\text{HCO}}_{3}^{-}$)
*k*_*d*_	first order decay rate (d^−1^)
*K*_*H,i*_	Henry's law coefficient for component *i* (kgCOD m^−3^ bar^−1^)
*K*_*I*_	concentration of inhibitor (polymer) giving 50% inhibition (kgCOD m^−3^)
*K*_*L*_	Langmuir affinity coefficient (m^3^ kgCOD^−1^)
*k*_*L*_*a*	dynamic gas-liquid transfer coefficient (d^−1^)
*k*_*m, ac*_	Monod maximum specific uptake rate (d^−1^)
*K*_*s, ac*_	Monod half saturation coefficient (kgCOD m^−3^)
*M*_*R*_	total solids mass inside the reactor (kgTSS)
*P*_*ch*4_	accumulated methane production (kgCOD kgVSS^−1^)
*p*_*gas*_	gas pressure (bar)
*p*_*gas,i*_	gas *i* partial pressure (bar)
*Q*_*e*_	adsorbent phase concentration of polymer after equilibrium (kgCOD kgTSS^−1^)
*Q*_*m*_	maximum adsorption capacity corresponding to monolayer coverage (kgCOD kgTSS^−1^)
*R*^2^	coefficient of determination (in linear models)
*S*_*i*_	concentration of soluble component *i* in the bulk liquid (kgCOD m^−3^)
*t*	time (s)
*t*_*c*_	time conversion factor (86,400 s d^−1^)
*V*	Volume of liquid in the reactor (m^3^)
*X*	concentration of VSS in the reactor (kgVSS m^−3^)
*X*_*ac*_	concentration of acetate degraders in the bulk liquid (kgCOD m^−3^)
*Y*_*ac*_	yield coefficient
*y*_*e, i*_	experimental observation *i* (SMA or AMP)
*y*_*m, i*_	model prediction of experimental observation *i* (SMA or AMP)
**Greek letters**	
β_*i*_	standardized regression coefficient for parameter *i*
γ^*k*^	collinearity index of the parameter subset *k*
υ_*i, j*_	stoichiometric coefficients of component *i* in process *j*
ρ_*j*_	rate of process *j* (kgCOD m^−3^ d^−1^^†^)
σ_θ_	standard deviation of estimators
θ	parameter subset for estimation
θ°	initial guess
$\hat{\theta }$	parameter estimators (optimal value)
**Subscripts**	
*ac*	acetate
*ch*4	methane
*co*2	carbon dioxide
*IC*	inorganic carbon
*n*2	nitrogen
*I*	inhibitor (polymer)

† For inorganic carbon, carbon dioxide and nitrogen the units are expressed in kmol instead of kgCOD.
