# Supplementary material for: Identification of the Methanogenesis Inhibition Mechanism Using Comparative Analysis of Mathematical Models
Source: Front Bioeng Biotechnol. 2019 May 8;7:93. doi: 10.3389/fbioe.2019.00093 (PMC6530379; doi:10.3389/fbioe.2019.00093)
Supplement: Supplementary file 1 [file Data_Sheet_1.pdf]

Supplementary Material

1 Supplementary experimental results and discussion

Supplementary Figure 1 shows that the polymer presented an inhibitory effect on the SMA. The SMA inhibition at a certain initial polymer concentration C_0 ($\text{SMA inhibition}|_{C_0}$) was estimated as the decrease in the SMA at C_0 ($\text{SMA}|_{C_0}$) with respect to the SMA without polymer addition ($\text{SMA}|_{C_0=0}$), and calculated as follows:

$$\text{SMA inhibition}|_{C_0} = 100\,\frac{\text{SMA}|_{C_0} - \text{SMA}|_{C_0=0}}{\text{SMA}|_{C_0=0}} \quad (1)$$

The SMA inhibitions were 24, 27, 40, 44, 53, 54, 65, and 69 % at 0.06, 0.11, 0.17, 0.23, 0.29, 0.34, 0.40, and 0.46 gCOD L^{-1} of polymer concentration, respectively. We performed a linear interpolation between the experimental results at 0.23 and 0.29 gCOD L^{-1} to estimate the polymer concentration that causes 50 % SMA inhibition, this was estimated to be 0.27 gCOD L^{-1}.

The SMA test were performed over a 10-day period. However, in the SMA tests with lower (or null) polymer concentration the acetate was rapidly degraded during a short experimental period, as shown in Supplementary Figure 1. After this period, not enough biogas was produced in the bottles to generate a new pulse in the AMPTS (the volume of the AMPTS cells was ~10 mL).

The one-way ANOVA test was used to study if there is a difference in the AMP final between batch tests performed at different polymer concentrations. The batch tests performed at concentrations of 0.40 and 0.46 g L^{-1} were not considered in the test because the methane was still being produced when we stopped the experiment. We obtained an F-test of 2.10 with a p-value of 0.114; thus, the p-value was above the α-level of 0.05. Therefore, the difference between the AMP final values is not statistically significant, i.e. it is unlikely that the AMP final changed with respect to polymer concentration.

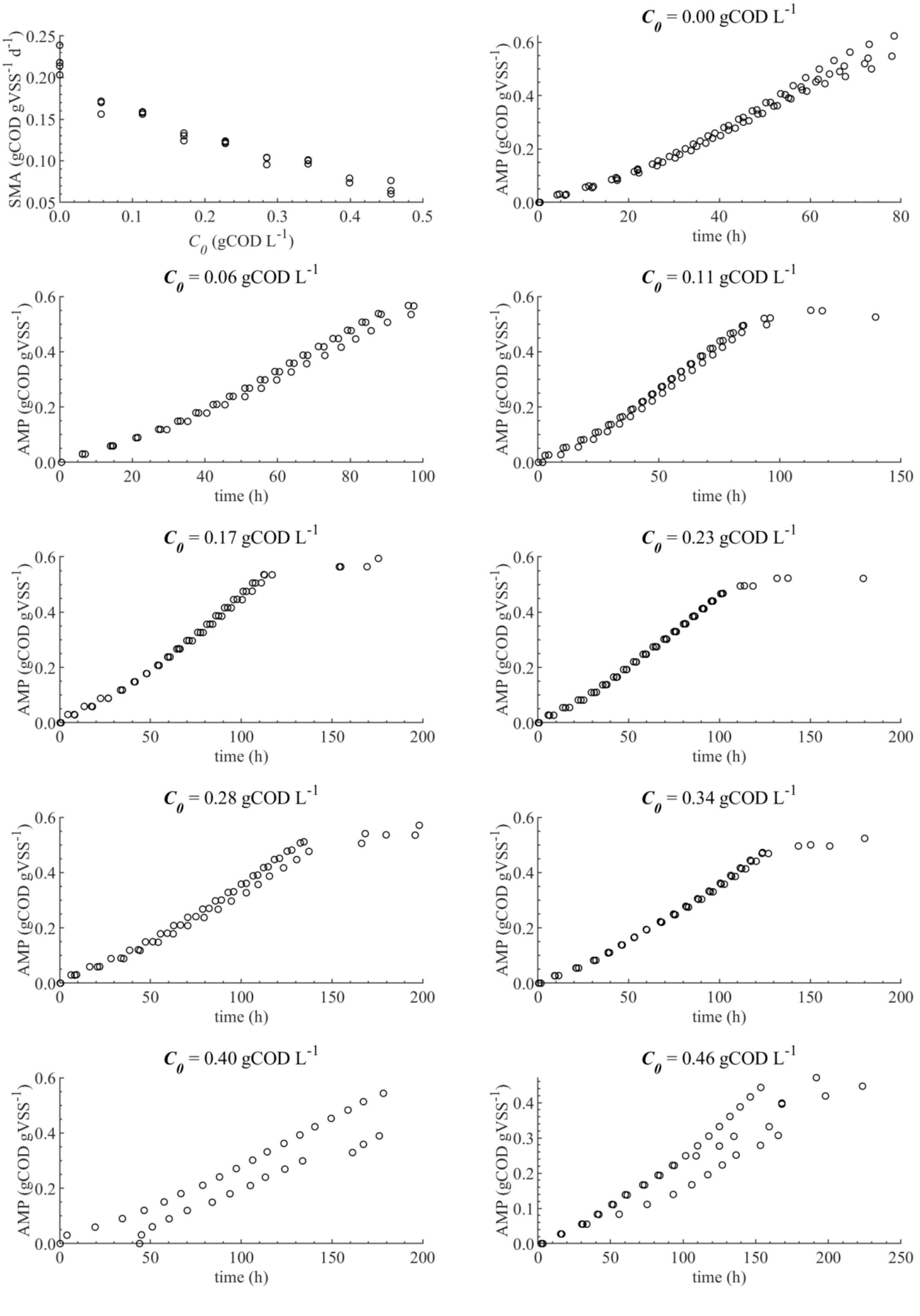

Supplementary Figure 1. Experimental results showing specific methanogenic activity (SMA) and accumulated methane production (AMP) over time obtained from batch test experiments using different concentrations of polymer (C_0).
